# Ultrahigh Temperature Flash Sintering of Binder-Less Tungsten Carbide within 6 s

**DOI:** 10.3390/ma14247655

**Published:** 2021-12-12

**Authors:** Huaijiu Deng, Mattia Biesuz, Monika Vilémová, Milad Kermani, Jakub Veverka, Václav Tyrpekl, Chunfeng Hu, Salvatore Grasso

**Affiliations:** 1 Key Laboratory of Advanced Technologies of Materials, Ministry of Education, School of Materials Science and Engineering, Southwest Jiaotong University, Chengdu 610031, China; Denghuaijiu9@outlook.com (H.D.); miladkermani.mk@my.swjtu.edu.cn (M.K.); chfhu@live.cn (C.H.); 2 Institute of Plasma Physics of the Czech Academy of Sciences, Za Slovankou 3, 182 00 Prague, Czech Republic; mattia.biesuz@unitn.it (M.B.); vilemova@ipp.cas.cz (M.V.); veverkaipp@cas.cz (J.V.); 3 Department of Inorganic Chemistry, Faculty of Science, Charles University, Hlavova 8, 128 43 Prague, Czech Republic; vaclav.tyrpekl@natur.cuni.cz

**Keywords:** tungsten carbide, ultrarapid consolidation, ultrahigh temperature flash sintering

## Abstract

We report on an ultrarapid (6 s) consolidation of binder-less WC using a novel Ultrahigh temperature Flash Sintering (UFS) approach. The UFS technique bridges the gap between electric resistance sintering (≪1 s) and flash spark plasma sintering (20–60 s). Compared to the well-established spark plasma sintering, the proposed approach results in improved energy efficiency with massive energy and time savings while maintaining a comparable relative density (94.6%) and Vickers hardness of 2124 HV. The novelty of this work relies on (i) multiple steps current discharge profile to suit the rapid change of electrical conductivity experienced by the sintering powder, (ii) upgraded low thermal inertia CFC dies and (iii) ultra-high consolidation temperature approaching 2750 °C. Compared to SPS process, the UFS process is highly energy efficient (≈200 times faster and it consumes ≈95% less energy) and it holds the promise of energy efficient and ultrafast consolidation of several conductive refractory compounds.

## 1. Introduction

Over the past few decades, ultrafast sintering methods have reached a high degree of sophistication. Shortening the consolidation time contributes to lower the energy consumed during the firing process. Starting from the 1970s, high heating rates have been exploited to enhance sinter ability while inhibiting the surface diffusion responsible for grain coarsening [1,2] active in the early stages of sintering [3]. Sintering techniques assisted by the application of an external electric field [4,5,6] allows ultrarapid densification and out-of-equilibrium material processing [7,8,9].

In 2010, Cologna et al. [10] discovered that 3 mol% yttrium-stabilized zirconia (3YSZ) can be sintered in a few seconds at 850 °C under an electric field of 120 V/cm. This process, with heating rates in the order of 104 K/min [11], was named Flash Sintering (FS) and its underlying mechanisms include: preferential overheating of the grain boundaries leading to thermal diffusion [12] or localized melting/softening [13,14], fast-heating enhanced densification [15,16] field-induced crystallographic defects nucleation, electric force interaction with the chemical potentials [17,18] and electrochemical effects [19,20,21].

Flash Sintering has been applied to several ceramics with different electrical conductivities: semiconductors [22,23,24], electronic conductors [25,26], ionic conductors [27,28,29], and composites [30,31,32,33]. Indeed, when considering good electric conductors, the flash can be reproduced at low temperature (or even at room temperature) but it requires the application of high electric current to induce sufficient Joule heating [34]. As a result, follow up activities have focused on the use of Spark Plasma Sintering (SPS) equipment to reproduce FS in conductive ceramics using low voltage (<10 V) and high current (a few kA) outputs. This technology was named Flash SPS (FSPS) and has been applied to different conductive ceramic systems [35,36], allowing densification in ≈20–100 s.

In parallel to the above mentioned advances in ceramics processing, Electrical Resistance Sintering (ERS) has been successfully applied to consolidate metals and cermets (i.e., WC composites with Co binder) allowing densification in less than one second [37,38,39]. However, these ultrafast sintering techniques are based on electrically insulating die in combination with copper or tungsten electrodes. As a results ERS remains only applicable to metals. The sample thermal shock generated by the contact between the sample and the cold die wall results inevitably in cracking of the ceramics [40].

Tungsten carbide (WC) is a highly refractory compound possessing excellent mechanical properties, high hardness, strength, wear resistance and chemical resistance to corrosive media. It is suitable for application such as cutting tools and abrasive materials [41,42,43]. Moreover, it is considered as a promising matrix for diamond composites with excellent wear resistance [44].

**Figure 1:**
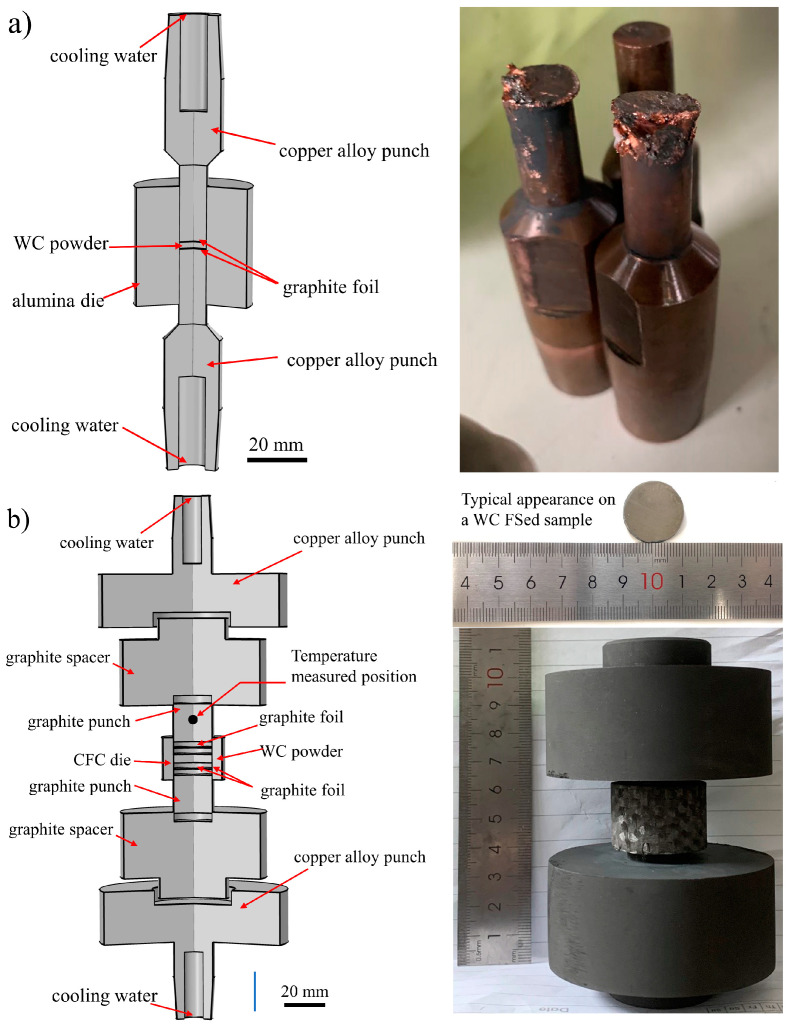
(**a**) Schematic representation of the alumina die and copper punches assembly commonly used in the ERS setup. The copper punches were damaged and the setup was unsuitable to consolidate binder-less WC. (**b**) Schematic and photograph of the newly developed experimental UFS set up with peak operating temperature well above 2500 °C. As shown in the inset, the samples were crack free.

To the best of authors’ knowledge, no attempt on flash-like densification (a few seconds) of binder-less WC has been made so far. Binder-less WC cannot be densified using ERS, the required temperature exceeding 2000 °C is incompatible with existing setup (i.e., cold die and metallic punches, see Figure 1a [45,46].

Herein, we report on the first flash consolidation of binder-less WC powder. A novel sintering apparatus was built and optimized to get near full densification of WC in a few seconds. Flash sintering is a pressure-less consolidation technique based on high voltage (typically greater than 50 V) and low current (<1 A) and it is not suitable to densify highly conductive and refractory carbides such as WC. In this work we proposed a novel pressure assisted flash sintering setup. In principle the technique is an upgrade of the existing ERS (Figure 1) with (maximum sintering temperature of 1500 °C), UFS extend the maximum temperature well above 2000 °C. The goal was to obtain crack-free, homogenous, and dense WC bulks with diameter of 20 mm.

Ultrahigh temperature flash sintering reflects an emerging research trend focused on ultra-fast and high energy efficient consolidation. It also opens opportunity to explore an ultrahigh temperature processing window (>2400 °C) under highly metastable processing conditions (i.e., heating and cooling rates > 10^3^ °C/min). This work further narrowed the sintering time compared to FSPS and it could pave the way for ultrafast sintering of several conductive ceramic systems.

## 2. Experimental Section

WC powder (Aladdin Industrial Corporation, Shanghai, China, MFCD00011464) with an initial particle size of ≈600 nm and purity >99% was used as the starting materials. The flash sintered WC samples were 20 mm in diameter weighed about 8 g. The sintering process was done with a modified spot-welding machine (DNB-350 KVA, China, MFBH-160/350-L/H) under a flowing Argon gas. The electric current was either applied in a single step or a stepwise profile (consisting of 3 steps). The DC high frequency rectified voltage was measured by an oscilloscope (UNI-T, UPO2104CS, Guangdong, China).

For comparison, a specimen with approximately similar relative density as UFSed compacts were also prepared using SPS (Ningbo Chenxin Weike Industrial Technology Co., Ltd., Ningbo, China). The SPS sample was heated using a heating rate of 100 °C/min, 5 min dwelling under a constant pressure of 20 MPa.

The sintered samples were mechanically polished to remove any carbon contamination. Density of the specimens were measured according to Archimedes’ principle. The microstructures were examined by a Scanning Electron Microscopy (SEM, Hitachi SU8220, Taiwan, China). Secondary electrons (SE) and backscattered electrons (BSE) were used to analyze the microstructure and grain size. Grain size was quantified using the by cut-off point method (GB/T 6394-2017), each sample was measured 5 times using a linear-weighted average. The hardness was measured on a polished surface by applying a force of 98 N for 15 s by a Vickers hardness tester machine (HVS-1000Z), the results were averaged over 5 valid indentations. To examine the composition of samples, X-ray Diffraction (XRD) was performed using a DX-2700BH diffractometer (10 kV and 5 mA) with Cu $K_{\alpha }$ = 1.54051 Å at a rate of 2 °/min between 20° to 80°, the quantitative Rietveld refinement of whole XRD profiles was performed in TOPAS v5 software.

The temperature was recorded every 0.5 s with an Infrared Pyrometer (Raynger 3i Plus) focused on the lateral side of graphite punch (temperature measured position as shown in Figure 1b). To obtain the real-time temperature of the specimen over the course of experiment, a Finite Element Model (FEM) was developed by means of the COMSOL^®^ software [47]. FEM was reproduced as detailed in Reference [48]. The electrical and thermal boundary conditions are shown in Figure S2, axial symmetric boundary condition was assumed along the central axis. An electrical power input was applied to the upper copper-alloy punch while the lower one was grounded, the lateral surfaces of graphite elements were assumed to be electrically insulated. The heat losses accounted for the contribution of radiation and convection assuming a transfer coefficient of 5 W/(m^2^K). The thermal and electrical conductivity, density and heat capacity employed in the model were temperature dependent.

## 3. Results and Discussion

Two different configurations were employed. In the first set of experiments, a sintering process was attempted following the ERS procedure [37]. The latter employs an alumina die and copper punches to force the electric current to only flow through the sample (Figure 1a). However, due to the high temperature and heat concentration at the WC/Cu interface the copper punches were partially molten and deformed (more details are discussed in the Supplementary Materials). Therefore, the experimental set up was modified (Figure 1b) by replacing the copper punches with graphite ones. The alumina die was replaced with a Carbon Fiber reinforced Carbon composite (CFC) with a low thermal inertia with mass of 9 g, well below both 111 g for SPS graphite die and 157 g for a Φ 10 mm ERS alumina die. The lower the mass the faster the heating. Furthermore, as shown in Figure 1b, two graphite spacers were also employed to avoid any damage to the copper electrodes of the spot-welding machine.

Table 1 summarizes the processing conditions and the relative density of the specimens. Initial attempts were performed by applying a straight 6 kA electric discharge using a single step for 6 s. By doing so, the pressed particles were ejected out of the die because of their low electrical conductivity. Presumably, such 6 kA discharge could not effectively flow across the powder (due to the presence of an oxide layer on the particles surface) [49] causing uneven heating. Under these conditions the voltage limit of 12 V was reached. It’s important to control the current flow in order to generate a homogeneous temperature distribution within the sample [50].

To allow densification in a short time, multi-step UFS process was proposed. It consisted of three steps including: preheating, sintering and cooling. In the preheating stage, the powder was sufficiently heated to allow decomposition of tungsten oxide and allow enough current flow (decomposition temperature of 1470 °C) [51]. In the second step, the current was relatively large, sufficient to allow a rapid consolidation. The third step allowed “slow” cooling to prevent thermal shock cracking.

**Figure 2:**
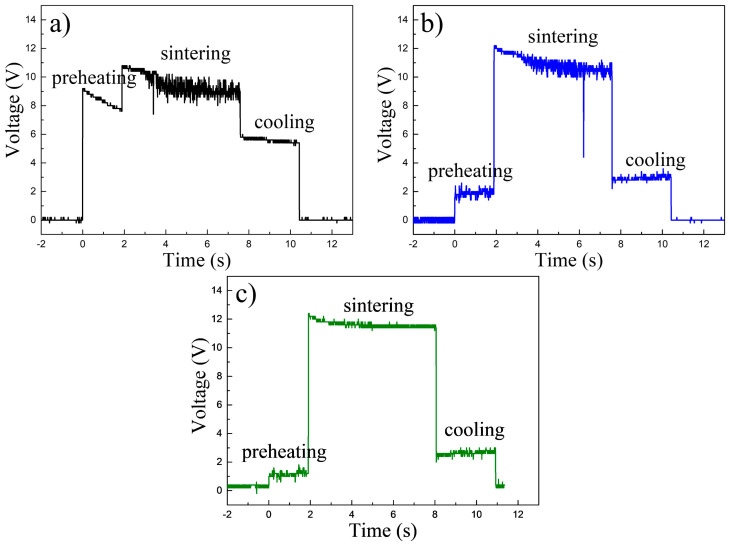
Voltage profiles with three processing steps of (**a**) sample UFS 1, (**b**) sample UFS 2 and (**c**) sample UFS 3.

**Figure 3:**
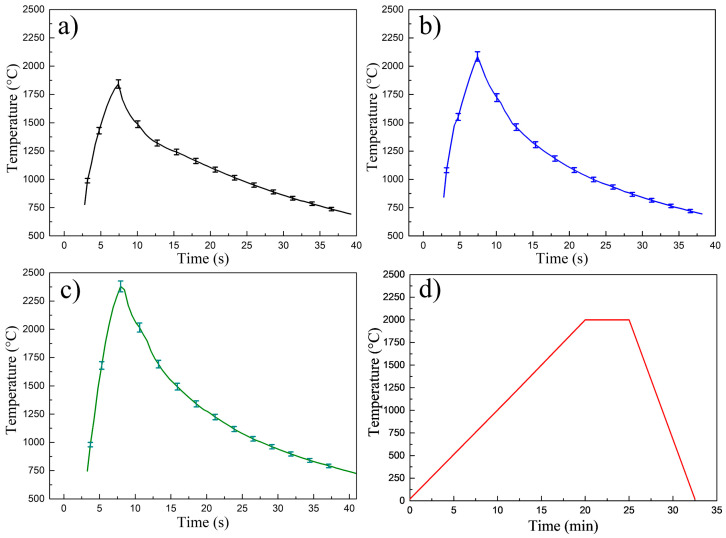
Experimentally recorded temperature profiles of (**a**) UFS 1, (**b**) UFS 2, (**c**) UFS 3 (temperature probing point T1 was 8 mm from the sample see Figure 4b, (**d**) SPS 4. Note the different time scales between SPS and UFS.

At present the equipment records the average current of each single step. The recorded voltages are well representative of the UFS process and they are shown in Figure 2. The experimentally recorded temperatures profiles are shown in Figure 3. The recorded current of samples UFS 1–3, for the second and third steps were 6 and 2.4 kA. During the initial step the current was rather unstable, the current flow was dependent on the conductivity of the loose powders. We attempted preheating (step 1) using an average voltage of 8.2, 1.9 and 1.2 V (Table 1). The results show that a low voltage allowed sufficient preheating to encourage an even current flow required for a homogeneous densification.

As the UFS process was very rapid, it was very challenging to accurately measure the sample temperature. The pyrometer recorded the temperature profile on a blind hole Φ 5 mm located at the center of the graphite punch 8 mm from the sample (Figure 3). The recorded temperature differed from the sample temperature due to the highly dynamic thermal gradients developed within the punch die assembly. The simulation of UFS process was employed to define the actual temperature of the sample.

**Figure 4:**
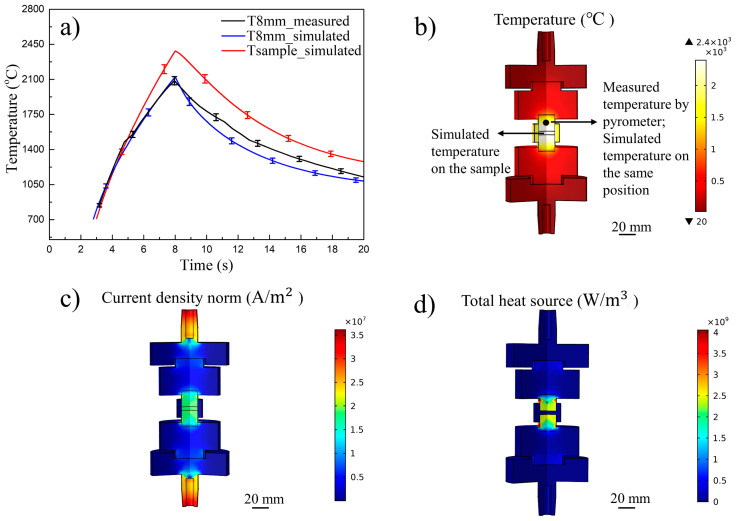
Electrothermal FEM distribution at the end of 6 kA discharge of sample UFS 2, (**a**) temperature profile, (**b**) temperature distribution, (**c**) current density distribution and (**d**) volumetric heat source.

The UFS process was simulated accounting for temperature, current and electrical power dissipation. The simulated temperature the gradient center to edge was below 100 °C. The hardness measured along the radial direction was within the scatter of the measurement. For example, the temperature curves measured and estimated in sample UFS 2 are shown in Figure 4a. One can observe that the simulated temperature at 8 mm from the sample well-matches the one measured with the pyrometer, thus validating the results of the simulations. The current and energy density distribution during the process are shown in Figure 4c,d, they reached values up to $2\times 10^{7}\;A/m^{2}$ and $4\times 10^{9}\;W/m^{3}$.

**Figure 5:**
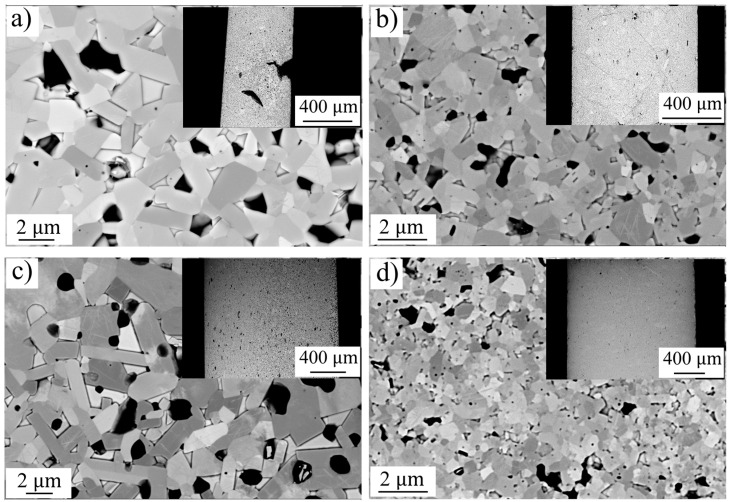
Back-scattered electron images with enhanced channeling contrast include (**a**) sample UFS 1, (**b**) sample UFS 2, (**c**) sample UFS 3 and (**d**) sample SPS 4.

For sample UFS 1, the high initial voltage of preheating step of 8.2 V contributed to the overheating and hot spots reflected in the inhomogeneous microstructure seen in Figure 5a. The exaggerated grain growth and the irregular morphology (see inset of Figure 5a) of the sample were possibly induced by the formation of an electrical arc. As expected, except from sample UFS1, the grain size followed a temperature dependence. As a result, the simulated temperature was not accurate because the FEM model assumed a homogenous heating. The sample UFS 2 showed a uniform microstructure, no compositional segregation was observed, the average WC grain size was 0.81 ± 0.09 μm as shown in Figure 5b. The simulated temperature profile measured on the punch was in good agreement with the experimental data, the peak temperature on sample reached 2441 °C and the resulting temperature distribution is shown in Figure 4b. The sample UFS 2 almost completely retained the δ−WC composition along with minor impurities of 2.1 wt.% $\beta^{\prime \prime }-W_{2}C$ and 2.3 wt.% graphite. Such impurities could be ascribed to the incomplete reaction of the free carbon contained in the starting powder. The sample UFS 3 showed exaggerated grain growth up to 1.6 ± 0.11 μm. The simulated sample peak temperature was as high as 2753 ± 54 °C (as shown in Figure 5c). Such excessive overheat resulted in formation of 10.5 wt.% $\beta^{\prime \prime }-W_{2}C$ phase due to the thermally activated carbon loss. For comparison, the sample SPS 4 was sintered by SPS process resulted in a morphology similar to sample UFS 2, average grain size of 0.6 ± 0.09 μm as shown in Figure 5d.

**Figure 6:**
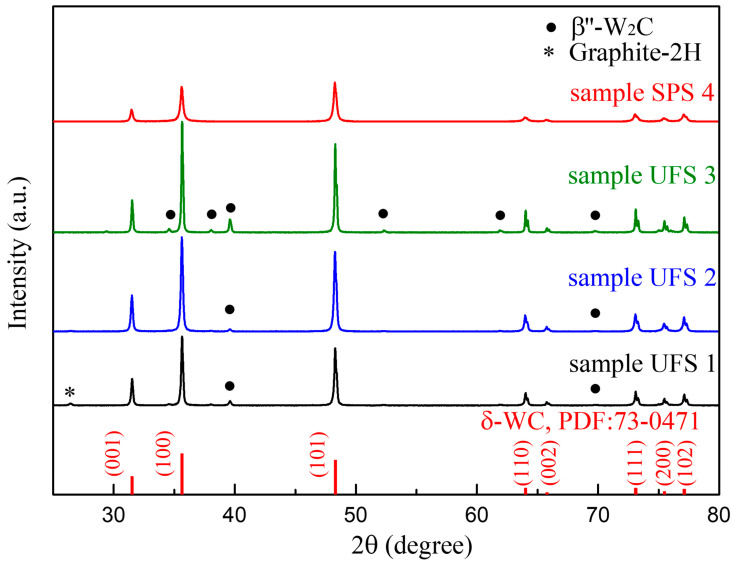
X-ray Diffraction (XRD) patterns of sample UFS 1, sample UFS 2, sample UFS 3 and sample SPS 4.

The composition of samples was analyzed with X-ray powder diffraction, as shown in Figure 6. The corresponding mass percentage of each phase along with relative density and hardness are shown in Table 2. Recalling the W-C phase diagram proposed by Kurlov and Gusev [52], the sample UFS 1 might have experienced the polymorphic transformation $\delta -\mathrm{WC}\to \gamma -{\mathrm{WC}}_{1-x}$ occurring 2735 ℃ and subsequently eutectoid decomposition upon cooling $\gamma -{\mathrm{WC}}_{1-x}\to \delta -\mathrm{WC}+C$. The latter seems in good agreement with the pronounced formation of graphitic carbon and the microstructural observation reflection the formation of hot spots seen in Figure 5a. On the contrary, the simulated temperature for samples UFS 2 (2383 ± 46) and UFS 3 (2753 ± 54 °C) might not have exceeded the polymorphic transformation responsible for the graphite formation. These results also suggest the possibility to use the polymorphic $\delta -\mathrm{WC}\to \gamma -{\mathrm{WC}}_{1-x}$ phase transformation to calibrate the FEM model. Samples were UFS 2 and SPS 4 had comparable hardness and were mostly composed of δ−WC. Sample UFS 3, despite the high relative density of 98.1% had a low Vickers hardness of 1576 because of the extensive formation of $\beta^{\prime \prime }-W_{2}C$ (reference Vickers hardness of 12 GPa ≈ 1224 HV) [53]. Analyzing the sample produced in the study, SPS process was about 200 times longer than UFS and it consumed about 40 times more energy. Therefore, UFS process is highly energy efficient.

Summarizing the results, the voltage in the preheating step had a significant effect on the homogeneity and quality of sintered bulks. The results show that voltage below 2 V allow sufficient preheating to facilitate the fabrication of uniform and dense samples. The results emphasize the significance of multiple step current profile to suit the rapid change of electrical conductivity experienced by the sintering powder during ultrafast sintering.

## 4. Conclusions

The Ultrahigh temperature Flash Sintering (UFS) demonstrated an ultrarapid (6 s) densification of binder-less tungsten carbide. The multistep process included a preheating step followed by a high current discharge. The pressed WC particles had limited conductivity, and a preheating step using a voltage below 2 V encouraged homogenous current flow. On the contrary, preheating step under a voltage exceeding 8 V led to inhomogeneous microstructures or even ejection of the powders from the die. The formation of hot spot and overheating of the samples resulted in the formation of $\beta^{\prime \prime }-W_{2}C$ and excessive grain growth, both leading to an undesired hardness degradation. Optimized UFSed samples were prepared by matching the electrical discharge profile with the rapid change of electrical conductivity of the sintering particles.

The UFS resulted in comparable hardness and phase purity of counterparts obtained using SPS. In addition, it brings significant advantages in terms of reduced energy consumption and efficiency of the process as most of the energy is directed to the sintering sample.

## Figures and Tables

**Table 1:** Experimental parameters (current, time, average voltage) and obtained relative density. The recorded current for the second and third steps were 6 and 2.4 kA.

Sample ID	First Step	Second Step	Third Step	Pressure (MPa)	Relative Density (%)	Remarks
UFS 0	Single step 6 s, 6 kA, 12 V			20	NA	WC powder ejected out from the die
UFS 1	2 s, 8.2 V	6 s, 9.5 ± 0.8 V	3 s, 5.6 V	20	91.3%	Inhomogeneous microstructure (hot spots)
UFS 2	2 s, 1.9 V	6 s, 10.9 ± 0.6 V	3 s, 2.9 V	20	94.6%	Homogenous
UFS 3	2 s, 1.2 V	6.5 s, 11.6 ± 0.2 V	3 s, 2.6 V	20	98.1%	Fairly homogenous but partially decomposed
SPS 4	heating rate of 100 °C/min, 5 min 2000 °C			20	96.7%	Homogenous

**Table 2:** Summary of processing conditions and properties of the samples prepared using SPS and UFS.

Sample ID	UFS 1 *	UFS 2	UFS 3	SPS 4
Average heating rate (°C/s)	228.5	277.3	316.6	1.67
Exp. Max Punch temperature (°C)	1843 ± 36	2086 ± 42	2441 ± 48	2000 ± 40
Sim. Maximum sample temperature (°C)	2058 ± 40 *	2383 ± 46	2753 ± 54	N/A
δ−WC (wt.%)	82.6	95.6	89.5	100
$\beta^{\prime \prime }-W_{2}C$ (wt.%)	5.2	2.1	10.5	0
Graphite-2H (wt.%)	12.2	2.3	0	0
Relative density (%)	91.3	94.6	98.1	96.7
Average grain size (μm)	1.4 ± 0.1	0.81 ± 0.09	1.6 ± 0.11	0.6 ± 0.09
Hardness (HV)	1923 ± 59	2124 ± 134	1576 ± 65	2057 ± 47
Energy consumed (MJ)	0.41 ± 0.05	0.42 ± 0.05	0.47 ± 0.06	14.14 ± 0.5

* Arcing was likely to occur and the temperatures were underestimated.

## Data Availability

Not applicable.

## Supplementary Materials

The following are available online at https://www.mdpi.com/article/10.3390/ma14247655/s1, Figure S1: X-ray Diffraction (XRD) pattern of the starting powder, Figure S2: Assumed FEM model’s boundary conditions for (**a**) electric and (**b**) thermal fields.
