# Supplementary material for: Ultrahigh Temperature Flash Sintering of Binder-Less Tungsten Carbide within 6 s
Source: Materials (Basel). 2021 Dec 12;14(24):7655. doi: 10.3390/ma14247655 (PMC8703299; doi:10.3390/ma14247655)
Supplement: Supplementary file 1 [file materials-14-07655-s001.zip › materials-1499197-supplementary.pdf]

Supporting Information

Ultrahigh Temperature Flash Sintering of Binder-Less Tungsten Carbide within 6s

Huaijiu Deng [1], Mattia Biesuz [2,3], Monika Vilémová [2], Milad Kermani [1], Jakub Veverka [2], Václav Tyrpekl [3], Chunfeng Hu [1] and Salvatore Grasso [1,*]

1 Key Laboratory of Advanced Technologies of Materials, Ministry of Education, School of Materials Science and Engineering, Southwest Jiaotong, Chengdu 610031, China
2 Institute of plasma physics of the Czech Academy of Sciences, Za Slovankou 3, 182 00 Prague, Czech Republic
3 Department of Inorganic Chemistry, Faculty of Science, Charles University, Hlavova 8, 2030 Prague, Czech Republic
* Correspondence: s.grasso@swjtu.edu.cn; Tel.: +86-184-8222-4962

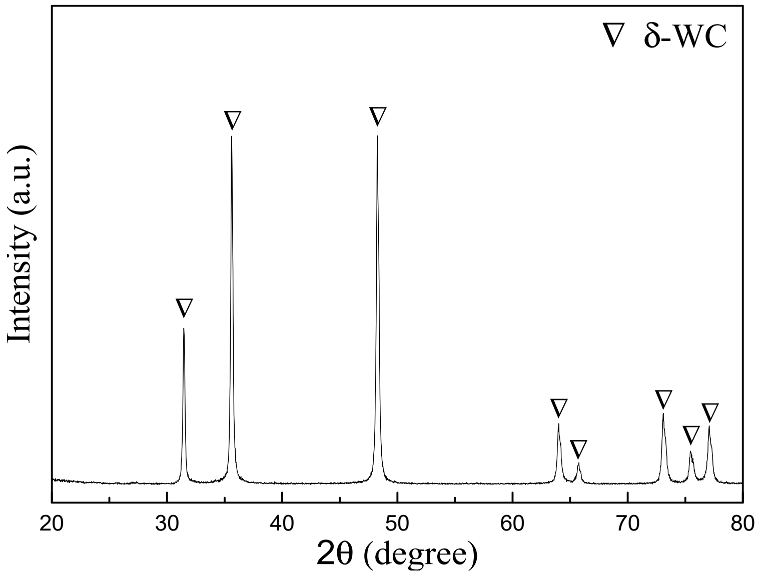

Figure S1. X-Ray Diffraction (XRD) pattern of the starting powder.

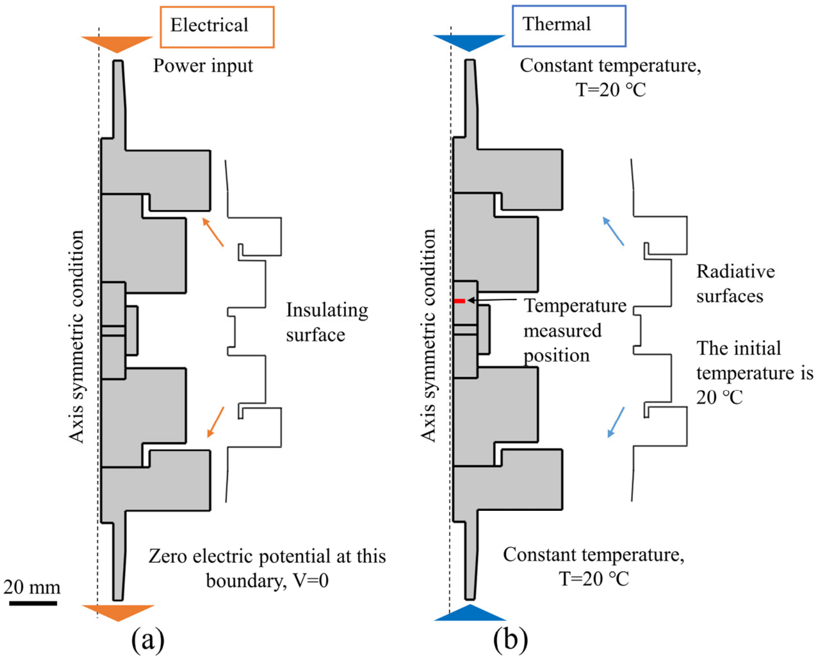

Figure S2. Assumed FEM model's boundary conditions for (**a**) electric and (**b**) thermal fields.

WC is a material "hard to densify". Tungsten carbide based ceramics are typically sintered in liquid phase (WC-Co or other binders) [1,2], where the metallic phase facilitates consolidation via liquid phase sintering and enhances the fracture toughness. Initial test were done using alumina dies as done in the previous investigation on capacitor discharge sintering [3,4] and Electric Resistance Sintering [5,6]. Unfortunately, due to the heat concentration, copper alloy punch was molten and heavily damaged as shown in Figure 1a.

References

1. Breval, E.; Cheng, J.P.; Agrawal, D.K.; Gigl, P.; Dennis, M.; Roy, R.; Papworth, A.J. Comparison between microwave and conventional sintering of WC/Co composites. *Materials Science and Engineering A* **2005**, *391*, 285–295, doi:10.1016/j.msea.2004.08.085.
2. Wang, X.; Xie, Y.; Guo, H.; van der Biest, O.; Vleugels, J. Sintering of WC-Co powder with nanocrystalline WC by spark plasma sintering. *Rare Metals* **2006**, *25*, 246–252, doi:10.1016/S1001-0521(06)60048-X.
3. Fais, A.; Leoni, M.; Scardi, P. Fast sintering of nanocrystalline copper. *Metallurgical and Materials Transactions A: Physical Metallurgy and Materials Science* **2012**, *43*, 1517–1521, doi:10.1007/s11661-011-0727-7.
4. Maizza, G.; Grasso, S.; Sakka, Y.; Noda, T.; Ohashi, O. Relation between microstructure, properties and spark plasma sintering (SPS) parameters of pure ultrafine WC powder. *Science and Technology of Advanced Materials* **2007**, *8*, 644–654, doi:10.1016/j.stam.2007.09.002.
5. Cannella, E. High precision tooling for electrical sintering of titanium and permanent magnets. *PhD Thesis - Technical University of Denmark* **2019**, 196, doi:978-87-7475-561-6.
6. Astacio, R.; Gallardo, J.M.; Cintas, J.; Montes, J.M.; Cuevas, F.G.; Prakash, L.; Torres, Y. Fracture toughness of cemented carbides obtained by electrical resistance sintering. *International Journal of Refractory Metals and Hard Materials* **2019**, *80*, 259–269, doi:10.1016/j.ijrmhm.2019.02.002.
